# MAAD: multidimensional antiviral antibody database

**DOI:** 10.1093/procel/pwaf106

**Published:** 2025-12-06

**Authors:** Yixin Li, Jinyue Wang, Chuziyue Zhang, Yuxia Zhang, Jie Deng, Han Zhang, Mingkai Li, Fan Wang, Xiangxi Wang

**Affiliations:** State Key Laboratory of Biomacromolecules, Institute of Biophysics, Chinese Academy of Sciences, Beijing 100101, China; University of Chinese Academy of Sciences, Beijing 100049, China; State Key Laboratory of Biomacromolecules, Institute of Biophysics, Chinese Academy of Sciences, Beijing 100101, China; University of Chinese Academy of Sciences, Beijing 100049, China; State Key Laboratory of Biomacromolecules, Institute of Biophysics, Chinese Academy of Sciences, Beijing 100101, China; University of Chinese Academy of Sciences, Beijing 100049, China; Changping Laboratory, Beijing 102206, China; Changping Laboratory, Beijing 102206, China; State Key Laboratory of Biomacromolecules, Institute of Biophysics, Chinese Academy of Sciences, Beijing 100101, China; University of Chinese Academy of Sciences, Beijing 100049, China; State Key Laboratory of Biomacromolecules, Institute of Biophysics, Chinese Academy of Sciences, Beijing 100101, China; University of Chinese Academy of Sciences, Beijing 100049, China; State Key Laboratory of Biomacromolecules, Institute of Biophysics, Chinese Academy of Sciences, Beijing 100101, China; University of Chinese Academy of Sciences, Beijing 100049, China; State Key Laboratory of Biomacromolecules, Institute of Biophysics, Chinese Academy of Sciences, Beijing 100101, China; University of Chinese Academy of Sciences, Beijing 100049, China; State Key Laboratory of Biomacromolecules, Institute of Biophysics, Chinese Academy of Sciences, Beijing 100101, China; University of Chinese Academy of Sciences, Beijing 100049, China; Changping Laboratory, Beijing 102206, China

**Keywords:** antibody database, sequence–structure–function integration, RNA viruses, rational antibody design

## Abstract

Antibodies have emerged as central components of therapeutic strategies against viral infectious diseases, functioning as key effectors in both prevention and treatment. While traditional antibody discovery has relied heavily on high-throughput screening, the field is now shifting toward rational antibody design, which requires integrative insights into sequence–structure–function relationships. However, existing resources provide a valuable foundation but remain limited in scope, highlighting the need for a standardized and well-annotated antibody database that integrates multidimensional features to further support systematic exploration, cross-pathogen comparison, and rational antibody design. Here, we introduce the Multidimensional Antiviral Antibody Database (MAAD; raabmd.org/raab/index), a curated platform dedicated to antibody, nanobody and single-chain variable fragment targeting three high-impact RNA virus families, Coronaviridae (SARS-CoV-1, SARS-CoV-2, MERS-CoV), Orthomyxoviridae (influenza virus), and Pneumoviridae (respiratory syncytial virus, human metapneumovirus), which were selected due to the large, high-quality datasets accumulated in recent years. MAAD further incorporates a suite of interactive analysis modules, including CDR and germline annotation, similarity-based sequence analysis, sequence-based clustering and structure-guided identification of antigen–antibody interface residues, complemented by per-site entropy and mutation rate profiling. These features enable in-depth exploration of antibody sequence characteristics, thereby facilitating functional and structural insights for rational antibody design. Together, by bridging antibody sequence, structure, and function, MAAD offers an open and standardized platform that advances comparative antiviral research and supports therapeutic antibody discovery.

## Introduction

Since the development of hybridoma technology, which enabled the generation of monoclonal antibodies (mAbs), mAbs have emerged as one of the most important classes of biotherapeutics, not only for the treatment of oncologic and autoimmune diseases, but also for combating viral infectious diseases (Köhler and Milstein, 1975; Pantaleo et al., 2022; Paul et al., 2024; Yasunaga, 2020). In particular, mAbs are promising prophylactic and therapeutic agents for viral infections due to their high specificity and immune-enhancing properties. Several antiviral mAbs have been approved by the US Food and Drug Administration (FDA). For example, palivizumab was the first FDA approved mAb for the prevention of respiratory syncytial virus (RSV) infection (Young, 2002); ibalizumab was authorized for the treatment of HIV-1 infection (Markham, 2018); and ansuvimab received approval in 2020 for the treatment of Ebola virus (EBOV) infection (Lee, 2021). During the COVID-19 pandemic, several mAbs, such as sotrovimab, casirivimab, and bamlanivimab, received Emergency Use Authorization (EUA) from the FDA (Deeks, 2021; Heo, 2022). While vaccines have played a central role in controlling the COVID-19 pandemic, mAbs have served as a vital countermeasure for high-risk populations, such as immunocompromised individuals, thereby underscoring their importance in mitigating emerging viral threats (Schmidt et al., 2024).

Building on these clinical advances, attention has increasingly shifted toward efficient strategies to accelerate mAbs discovery and optimization. Traditional antibody discovery has relied heavily on high-throughput screening (Mahdavi et al., 2022). In contrast, contemporary efforts increasingly emphasize rational antibody design, which relies on a comprehensive understanding of sequence, structure, and function relationships to enable precise optimization of affinity, specificity, stability, and breadth. Importantly, such insight not only enables the engineering of antibodies with desired properties, but also advances antibody-based vaccinology (Lanzavecchia et al., 2016). Specifically, antibody-based vaccinology aims to overcome the limitations of traditional vaccine approaches by designing novel immunogens based on structural characterization of antigen–antibody complexes (Pantaleo et al., 2022). By identifying protective epitopes and masking immunodominant but non-neutralizing regions, this approach focuses the immune response on functionally critical targets, thereby enhancing vaccine efficacy. This strategy has been successfully applied in the design of immunogens targeting conserved neutralizing epitopes on influenza hemagglutinin (HA) (Weidenbacher and Kim, 2019). It has also been utilized in SARS-CoV-2 vaccines that display receptor-binding domains (RBDs) in a highly immunogenic array and exhibit a lower antibody binding: neutralizing ratio (Walls et al., 2020). The above-mentioned successes highlight the dual role of mAbs as both therapeutic agents and blueprints for vaccine design. Meanwhile, to address the growing demand for mAb discovery, artificial intelligence (AI) has emerged as a powerful tool to accelerate their identification and optimization (Lou et al., 2023). However, the performance of AI-driven approaches critically depends on large-scale, standardized training data that systematically connect sequences and structures to their functional properties. To support rational antibody design, antibody-based vaccinology, and AI-driven antibody discovery, there is an urgent need for a standardized, well-annotated antibody database that integrates sequence, structure, and function data into a coherent platform.

**Figure 1.**
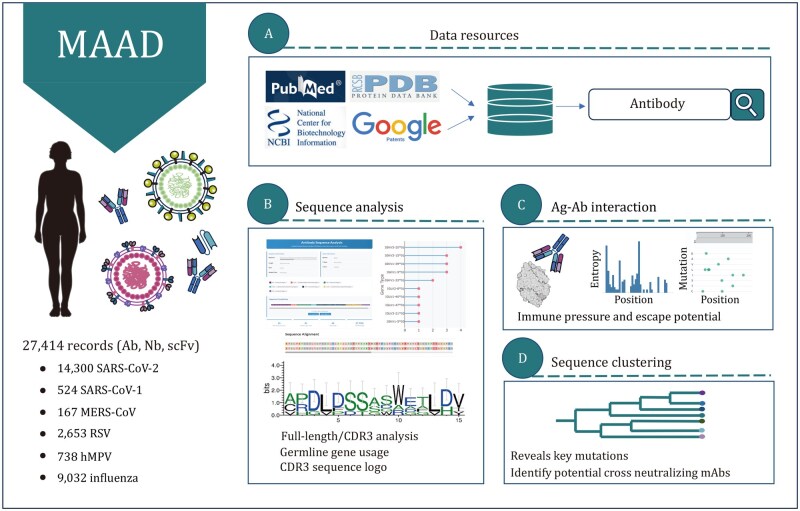
**Overview of MAAD and its functional modules**. (A) Antibody, nanobody, and scFv entries were systematically collected from multiple sources (Berman et al. 2000; Benson et al. 2011; NCBI Resource Coordinators 2017; patents.google.com), including peer-reviewed publications, patents, and clinical sources, and integrated into the unified MAAD database with standardized annotations. (B) The platform supports interactive sequence analysis, including CDR annotation, V/J germline gene assignment, and similarity-based searches using full-length or CDR sequences. (C) For antibodies with resolved structures, MAAD provides detailed interface residue annotations, coupled with per-site Shannon entropy and mutation frequency based on viral sequence diversity. (D) A sequence-based clustering and phylogenetic tree construction module is implemented to group antibodies and nanobodies.

**Figure 2.**
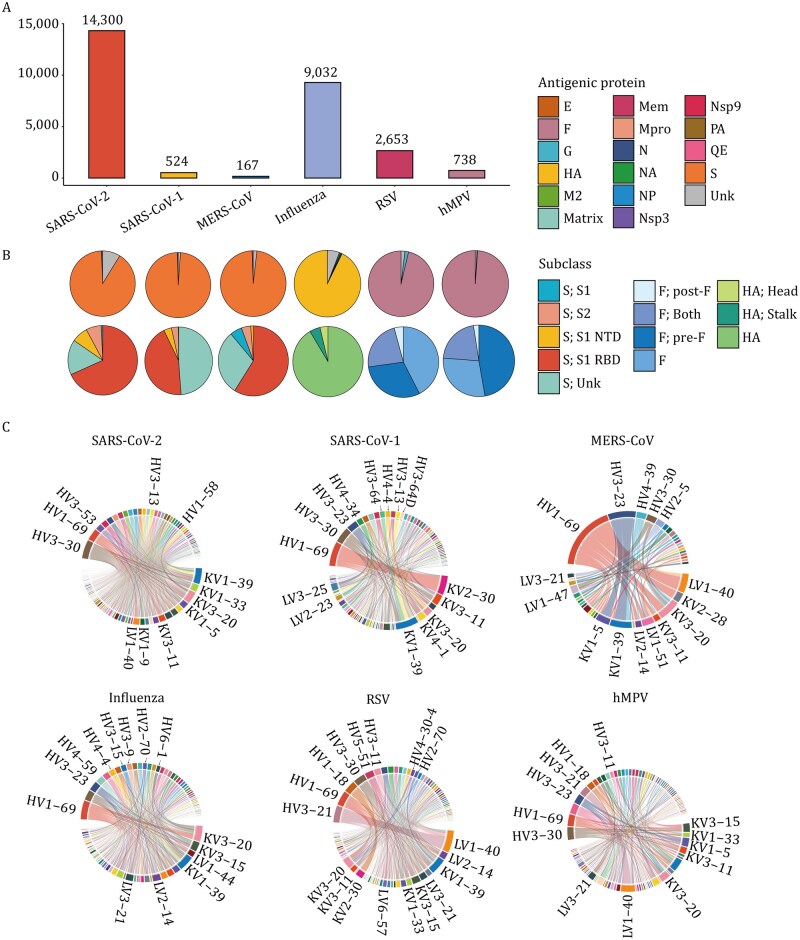
**MAAD statistics and antibody gene usage profiles**. (A) The number of curated entries primarily derived from three viral families, including *Coronaviridae* (SARS-CoV-1, SARS-CoV-2, MERS-CoV), *Orthomyxoviridae* (influenza A/B), and *Pneumoviridae* (RSV, hMPV). (B) Pie charts in the first row illustrate the distribution of antigenic protein targets. S, spike protein; N, nucleocapsid protein; Mem, membrane protein; E, envelope protein; Nsp, non-structural protein; Mpro, main protease; HA, hemagglutinin; NA, neuraminidase; NP, nucleoprotein; M2, matrix protein 2; F, fusion protein; G, attachment protein; QE, quaternary epitope; Unk, unknown. Pie charts in the second row illustrate the distribution of dominant antigenic protein subcategories across different viruses. For coronaviruses (SARS-CoV-1, SARS-CoV-2, and MERS-CoV), spike-specific antibodies are further classified as: S1, S2, S1-RBD (receptor-binding domain), S1-NTD (N-terminal domain), and unknown regions. “S1” refers to antibodies mapped to the S1 subunit but lacking finer resolution to assign them to RBD or NTD; “Unknown” denotes antibodies known to bind S protein but with no specific subunit (S1/S2) information available. For RSV and hMPV, the F protein is categorized into prefusion (pre-F), postfusion (post-F), or cross-binding to both. For influenza viruses, hemagglutinin (HA)-specific antibodies are subdivided into those targeting the HA head or stalk regions. (C) Circos plots showing the top 10 IGHV-IGLV pairings observed across antigen-specific human-derived antibodies (only gene pairs with frequencies >1 shown).

One of the major challenges in antibody engineering is to elucidate the relationships among sequence, structure, and function that govern antibody specificity and breadth. Although existing antibody databases have provided valuable resources to the field (Dunbar et al., 2014; Olsen et al., 2022; Raybould et al., 2021), there remains a need for a platform that comprehensively integrates sequence, structural, and functional annotations across diverse viral pathogens (Table 1). To address this gap, we introduce a multidimensional database of antiviral antibody (MAAD), which integrates 27,414 antibody, nanobody, and single-chain variable fragment (scFv) entries (Figs. 1 and S1A). MAAD focuses on antibodies targeting three high-impact RNA virus families including Coronaviridae (severe acute respiratory syndrome coronavirus 1 [SARS-CoV-1], severe acute respiratory syndrome coronavirus 2 [SARS-CoV-2], Middle East respiratory syndrome coronavirus [MERS-CoV]), Orthomyxoviridae (influenza virus), and Pneumoviridae (RSV, human metapneumovirus [hMPV]), due to the large, high-quality datasets accumulated in recent years (Fig. 2A). These antibodies benefit from standardized binding and neutralization assays, making them ideal for building a comprehensive and functionally annotated database.

**Table 1.** Comparison of MAAD with existing antibody databases.

**Feature**	CoV-AbDab (Raybould et al., 2021)	SAbDab (Dunbar et al., 2014)	OAS (Olsen et al., 2022)	MAAD
Scope	Ab/Nb/scFv against CoVs	Ab/Nb/scFv with resolved structures	Broad	Ab/Nb/scFv against several antigens
Sequence type	Paired aa sequences	Paired aa sequences	Unpaired and paired aa and nt sequences	Paired aa and nt sequences
Functional annotation	Binding, neutralization	Binding (with resolved structures)	None (repertoire only)	Binding, neutralization
Interface residues annotation	None	None	None	Antigen–antibody interface residues annotated
Virological data	None	None	None	Per-residue Shannon entropy and mutation rate at interface positions
Interactive modules	Basic search	Basic search and structural viewer	Basic search	Basic search, CDR and germline annotation, antigen–antibody interfaces, structural viewer, phylogenetic clustering

aa, amino acid; Ab/Nb/scFv, antibody/nanobody/single-chain variable fragment; nt, nucleotide.

To further facilitate user-driven analysis, MAAD also integrates a suite of interactive analysis and visualization modules for complementarity-determining regions (CDRs) and germline annotation, similarity-based entry search, identification of antigen–antibody interface residues, per-site Shannon entropy and mutation analysis, as well as phylogenetic clustering of antibody sequences. By linking antibody sequence, structure, and function through integrated analysis and visualization modules, MAAD serves not only as a comprehensive data resource but as a versatile platform that facilitates rational antibody and vaccine design.

## Results

### Comprehensive functional annotation of entries in MAAD

Currently, MAAD integrates 27,414 standardized antibody, nanobody, and scFv entries compiled from 805 peer-reviewed publications and 140 patents. These entries cover six high-impact respiratory pathogens across three viral families, including *Coronaviridae* (SARS-CoV-1, SARS-CoV-2, MERS-CoV), *Orthomyxoviridae* (influenza A and B), and *Pneumoviridae* (RSV, hMPV) (Fig. 2A). Among the 27,414 entries, approximately 17,600 entries are experimentally annotated with detailed functional data such as binding and/or neutralization. The remaining entries, although lacking direct experimentally validated functional evidence such as binding or neutralization data, are primarily derived from antigen-specific B cell repertoires. These sequence-only entries retain complete variable region sequences and serve as a valuable resource for clonal lineage inference and the training of machine learning models for antibody function prediction.

Each entry is annotated with key metadata, including the published name of the antibody, nanobody, and scFv, antigen target, biological or synthetic origin (e.g., infected human, immunized mouse, engineered), and the experimentally validated antigen-binding and/or neutralization specificities (Table S1). Full-length variable region sequences of each entry are numbered using ANARCI (Dunbar and Deane, 2016), which employs hidden Markov models to align input sequences to pre-numbered germline references. CDRs are annotated based on three standardized numbering schemes: the international IMGT (Lefranc et al., 2003), Kabat (Kabat and Wu, 1971), and Chothia (Chothia and Lesk, 1987). When available, nucleotide sequences are included, along with corresponding GenBank (Benson et al., 2011) accession numbers. Structural information is linked directly to corresponding Protein Data Bank (PDB) (Berman et al., 2000) entries. In addition, all records are cross-referenced to their original literature sources via PubMed ID (PMID) or patent number and publication date.

Specifically, the majority of entries are directed against SARS-CoV-2 (WT = 12,057; alpha = 734; beta = 1,161; gamma = 735; delta = 1,190; epsilon = 2,469; omicron = 3,123), reflecting the intensive research focus during the COVID-19 pandemic. The database additionally comprises entries targeting MERS-CoV (*n *= 70), SARS-CoV-1 (*n *= 2,208), influenza viruses (influenza A = 1,257; influenza B = 248), RSV (RSV-A = 1,479; RSV-B = 910), and hMPV (hMPV-A = 291; hMPV-B = 271) (Fig. S1B). Notably, entries are not mutually exclusive across antigens, as individual antibodies may have been tested against multiple targets. Based on all antibody and nanobody entries collected in the MAAD, we analyzed the distribution of viral protein targets (Fig. 2B). For all three coronaviruses (SARS-CoV-1, SARS-CoV-2, and MERS-CoV), the spike protein is the predominant target, with the RBD being the most investigated domain (Fig. 2B). In the case of influenza virus, HA is the predominant target of antibody binding, accounting for over 93% of entries, while antibodies against other viral components such as nucleoprotein (NP) and neuraminidase (NA) are extremely rare (<1%) (Fig. 2B). For Pneumoviridae members RSV and hMPV, most antibodies are directed against the fusion (F) protein. For RSV, over 96% of antibodies bind to F protein, with approximately 2% targeting the attachment glycoprotein (G) (Fig. 2B). In hMPV, the vast majority of antibodies (99%) target the F protein, whereas only a small fraction recognizes the matrix protein (Fig. 2B). Notably, we observed that for both RSV and hMPV, F-specific antibodies mainly recognize the prefusion conformation or exhibit cross-reactivity, whereas postfusion-specific antibodies are rare (Fig. 2B). MAAD also incorporates 67 clinically evaluated therapeutic antibodies from regulatory documents and published clinical studies. These entries, which are readily accessible on the download page, cover antibodies targeting viral antigens represented in MAAD, including SARS-CoV-2, MERS-CoV, RSV and influenza, thereby providing important benchmarks for therapeutic development. By including antibodies that have advanced into clinical use, MAAD offers real-world evidence of efficacy and safety, which enables comparative analyses with preclinical candidates.

### Interactive exploration and visualization for antibody sequence analysis

To facilitate intuitive exploration and functional analysis of each entry, MAAD implements a set of interactive modules for data query, visualization, and sequence analysis. In the search and filtering module, users can perform name-based searches to quickly locate specific entries of interest, or use virus-based searches to identify entries derived from B cells exposed to a particular virus through infection or immunization. Additional filters are provided for biological origin (e.g., human, murine, camelid, synthetic), V/J germline gene usage, PDB or project-specific identifiers (Fig. S4A). Each matched entry links to a detailed information page including comprehensive metadata such as targeted epitopes, full-length variable region sequences, CDR annotations across IMGT, Kabat, and Chothia schemes, somatic hypermutation (SHM), structural information (PDB), and available binding or neutralization profiles. Meanwhile, references are hyperlinked, allowing users to trace each record back to its original publication or patent source for straightforward verification (Fig. S4A).

**Figure 3.**
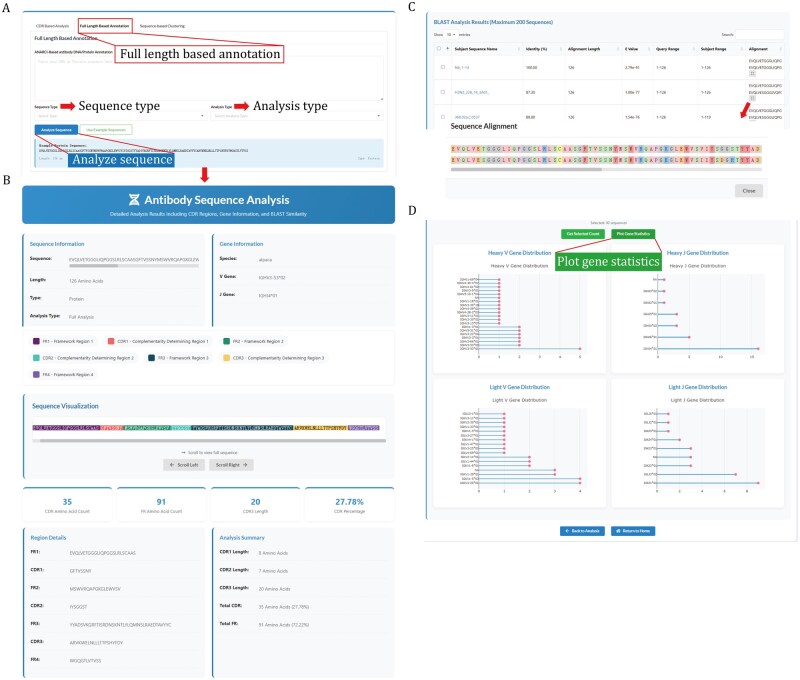
**Detailed page of the sequence analysis module**. (A) Interactive interface for Full-length-based similarity search. (B) Full-length antibody sequence analysis results with V/J germline assignment and framework/CDR annotation based on the IMGT scheme. (C) Results of sequence-based similarity searches. (D) Statistical summary of selected entries, with dot plots illustrating germline gene distributions.

In addition to metadata-based queries, MAAD also supports both “Full-length-based” and “CDR-based” similarity searches within the Analysis module, enabling users to input custom antibody variable region sequences and retrieve matched entries (Figs. 3A and S4B). Two analysis modes are available: (i) Full-length-based analysis mode: the input sequence is first processed through ANARCI, which returns CDR annotations and corresponding V/J germline genes. The annotated query sequence is then aligned against all MAAD entries using BLAST (Altschul et al., 1990). Matched entries are interactively selected by the user, and upon clicking the “Analyze” button, the selected sequences are summarized with V/J gene usage dot plots to assess germline convergence or divergence patterns (Fig. 3B). (ii) CDR-based analysis mode: users may focus on specific CDR regions (e.g., CDR3) as input. The search algorithm matches sequences of identical length and detects similar or embedded motifs using N-Gram indexing and LIKE-based search. Selected CDR3 sequences can then be visualized as sequence logo plots generated by WebLogo (Crooks et al., 2004), thereby highlighting conserved and variable residues (Fig. S4B). Associated V/J gene dot plots are also provided to reveal lineage biases within matched antibody repertoires.

To support interactive analysis of user-provided sequences, MAAD’s Statistics module offers an overview of germline gene usage patterns across the entire database in its genotype distribution mode. By profiling V and J gene pairings in both heavy and light chains, we revealed distinct patterns across viral targets (Figs. 2C and S3). Among human-derived antibodies, certain IGHV genes such as IGHV3-30 and IGHV1-69 were broadly utilized across multiple viruses, however, the associated light-chain partners exhibited considerable diversity (Fig. 2C). For example, IGLV1-40, IGKV3-11, and IGKV3-20 frequently paired with IGHV1-69 in SARS-CoV-2 antibodies, whereas IGKV2-30 was the predominant light-chain partner for IGHV1-69 in SARS-CoV-1and IGHV1-69/IGKV3-20 pairing was highly enriched in influenza. In contrast to the broad usage of IGHV1-69 and IGHV3-30, RSV antibodies exhibited a distinct preference for IGHV3-21, which was rarely observed in other viruses. Interestingly, IGHV3-21 was also detected in a subset of hMPV-responsive antibodies, suggesting a potential cross-reactive germline signature shared among pneumoviruses. Together, these findings highlight both conserved and pathogen-specific germline biases that may underlie differential antibody recognition and cross-reactivity across respiratory viruses (Fig. 2C). Overview of CDR3 length distributions was also provided in MAAD’s Statistics module. Heavy-chain CDR3s generally displayed broader variability than light chains, with light-chain CDR3 lengths concentrated around 9–11 amino acids, whereas heavy-chain CDR3s were distributed more broadly, ranging from 10 to 23 amino acids (Fig. S2A). In terms of SHM, the majority of antibodies carried fewer than 40 nucleotide substitutions (Fig. S2B), whereas those with extremely high substitution frequencies likely originated from artificially affinity-matured antibodies generated by phage display or other engineering approaches. The detailed page of each entry additionally reports per-region SHM profiles, including mutations in framework regions 1/2/3 and CDR1/2, along with counts of replacement versus silent substitutions (Fig. S4A). Together, these analyses highlight both conserved and virus-specific features of antibody repertoires.

### Integration and annotation of antigen–antibody interaction profiles

**Figure 4.**
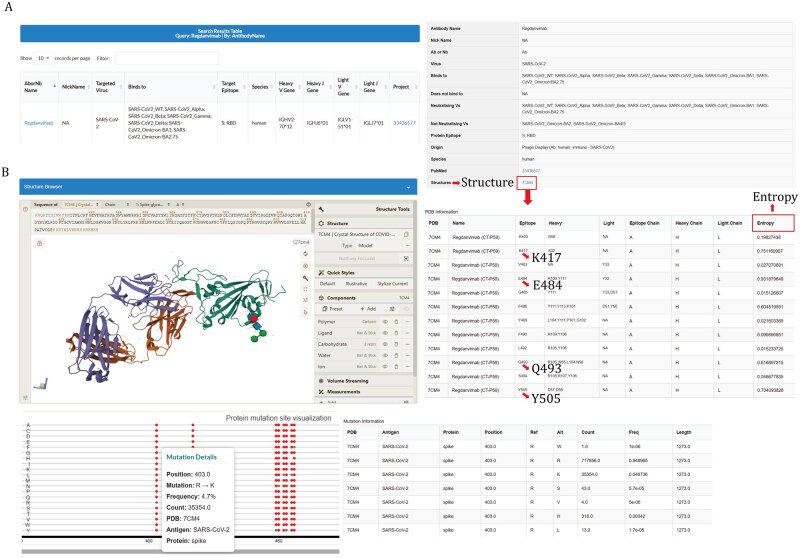
**Detailed page of antigen–antibody complex interaction profiles**. (A) Example workflow illustrating how resolved antigen–antibody complex structures are identified. (B) Detailed page of resolved antigen–antibody structures and Shannon entropy of interface residues for each pathogen (top). Dot plot representation of mutation frequencies at interface residues across pathogens (bottom).

Given the continuous emergence of viral variants, structural information is essential for dissecting antibody–antigen interactions. MAAD currently integrates 1,394 resolved antigen–antibody complex structures, and each entry is linked to its corresponding PDB. Users can access these data through the Search module by entering PDB. Each matched entry is linked to an interactive structure page where the antigen chains and the antibody chains are identified and labeled, respectively (Fig. 4A). To characterize binding interfaces, interface residues were defined as antigen or antibody residues that have at least one interatomic contact within 4.5 Å. The web page provides a 3D interactive viewer that displays the complex structure with the interface residues in a table. Users can toggle the interface view, and hover over residues to display residue-level information such as amino acid identity and position number. This visualization allows users to directly cross-reference the 3D structure with the interface residue table, facilitating intuitive exploration of specific amino acid interactions at the binding interface.

**Figure 5.**
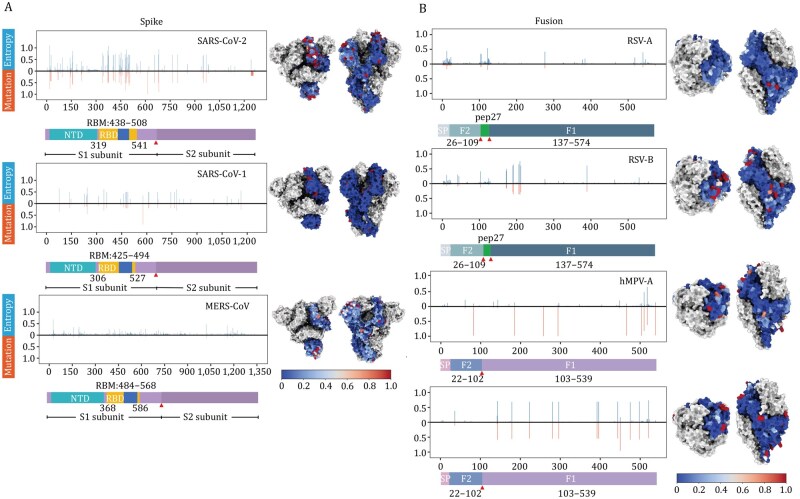
**Viral entropy and mutation analysis**. (A and B) show site-specific sequence variability and mutational landscape of viral antigens. Bar plots display per-residue Shannon entropy (blue) and mutation frequency (orange), calculated based on aligned viral sequences relative to a reference strain (A: spike protein; B: fusion protein). The bars below represent the spike protein (A) and the fusion protein (B), with major functional regions highlighted. The red arrow indicates the cleavage site. Corresponding 3D antigen structures are colored by normalized Shannon entropy values to visualize surface variability. Two structural orientations are shown: top view (left) and side view (right). Structural models were obtained from PDB entries 7K90, 5XLR, 7YN0, 8WZ3, and 8VT2.

To assess antigenic variability at antigen–antibody interfaces, MAAD annotates interface residues with Shannon entropy scores, reflecting site-specific amino acid diversity (Figs. 5A, 5B, S5A and S5B). The Shannon entropy scores are available in the Statistics module. For coronaviruses, entropy was calculated for spike protein, which comprises the majority of resolved antigen–antibody complexes (Figs. 2B and 5A). In SARS-CoV-2, elevated entropy was observed within spike residues 319–541 (Fig. S5C), corresponding to the RBD and receptor-binding motif (RBM), well-known hotspots of immune pressure (Jian et al., 2025). By contrast, MERS-CoV spike displayed relatively uniform entropy, while the limited number of sequences restricted the analysis of SARS-CoV-1. For RSV and hMPV, entropy and mutation rate profiles were assessed independently for the F and G proteins from both subtype A and B strains (Figs. 5B and S5B). The F protein of both RSV-A and RSV-B displayed generally low variability, with minor entropy peaks observed in the N-terminal regions around residues 0–25 (signal peptide) and 110–135 (p27). However, RSV-B showed pronounced peaks of entropy and mutation frequency particularly at residues 42, 45, 172, 173, 190, 191, 206, 209, 211 and 389, whereas RSV-A displayed notable variability hotspots at residue 276, 377, 284, 518 and 540 (Fig. 5B). In contrast, the RSV G protein was markedly more variable (Fig. S5B), with both RSV-A and RSV-B exhibiting extensive entropy peaks and frequent mutations across the mucin-like regions, while the central conserved domain (CCD) remained relatively stable (Fig. S5C). For hMPV, the F protein of both subtypes also exhibited overall low variability, with sporadic peaks of entropy and mutations primarily within the F1 subunit (Fig. 5B). The hMPV G protein exhibits variability in its mucin-like domains, similar to the RSV G protein (Fig. S5B and S5C). These observations highlight that while both RSV and hMPV F proteins are generally conserved with subtype-specific differences in variability distribution, their G proteins exhibit extensive sequence diversity. For influenza virus, the major surface glycoprotein HA is critical for facilitating virus entry and infection of host cells and exhibits relatively low sequence conservation across strains owing to its antigenic diversity and rapid evolution (Wilson et al., 1981; Wu and Wilson, 2020). To investigate sequence variability, we collected HA sequences from human-derived strains of major public health concern (H1N1, H3N2, H5N1, H7N9, and two influenza B lineages) (Bi et al., 2024; Fasanmi et al., 2017; Su et al., 2017) and performed Shannon entropy analyses separately. Consistent with previous research, Peaks of sequence variability were predominantly concentrated within HA1, whereas HA2 showed overall lower entropy across all examined influenza subtypes, except for Yamagata, supporting HA1 as the major target of antigenic drift (Fig. S5A). This pattern reinforces the concentration of sequence variability in HA1, whereas HA2 remains comparatively conserved (Fig. S5A and S5C).

### Sequence-based clustering and tree construction of antibodies

**Figure 6.**
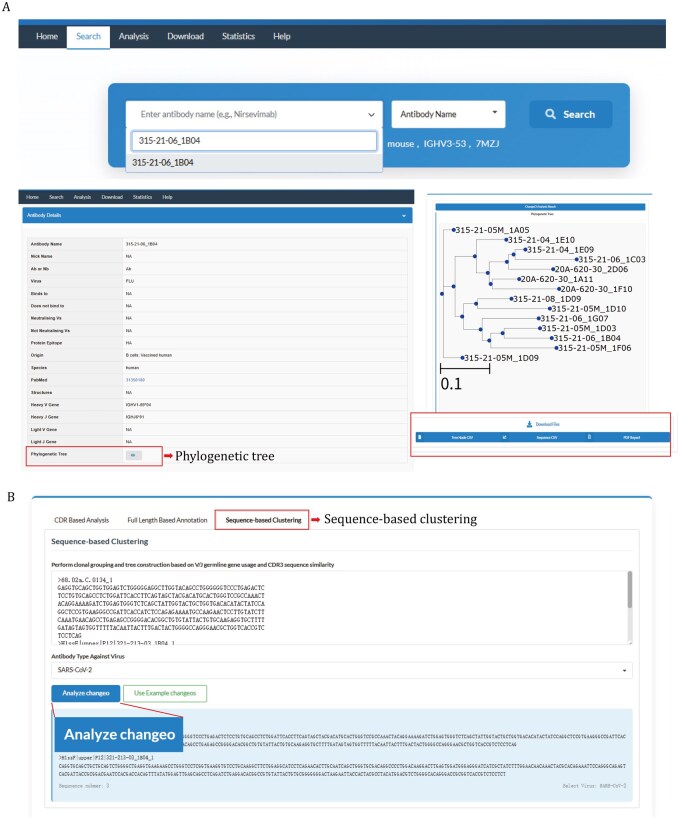
**Detailed page of sequence-based phylogenetic clustering and tree construction**. (A) Example workflow illustrating how to view and download precomputed phylogenetic trees that integrate both functionally validated and uncharacterized entries across the database. (B) Interactive interface for user-submitted sequence phylogenetic clustering.

With the rapid growth of antibody repertoire data generated by next-generation sequencing (NGS), a substantial portion of NGS-derived sequences remain experimentally unvalidated, lacking direct evidence of antigen binding or neutralization. To address this challenge, MAAD implements a “sequence-based clustering” mode within the Analysis module, which integrates sequence-based clustering and phylogenetic reconstruction to group functionally unvalidated antibodies alongside annotated ones, thereby facilitating comparative analysis and hypothesis generation regarding potential functional similarity (Fig. 6A and 6B). This module enables users to perform clonal grouping based on V/J germline gene usage and CDR3 sequence similarity. For heavy-chain sequences, clonotype assignment was inferred with the Change-O toolkit (Gupta et al., 2015), based on germline annotations to cluster similar sequences. Within each assigned cluster, sequences were aligned using MAFFT (Katoh et al., 2002), and maximum-likelihood phylogenetic trees were generated by IQ-TREE (Nguyen et al., 2015) with appropriate evolutionary models to illustrate the phylogenetic relationships among clonally related antibodies (Fig. S6A).

MAAD supports two modes of phylogenetic analysis: (i) user-driven phylogenetic tree reconstruction, in which user-uploaded, FASTA-formatted sequences are integrated with MAAD sequences to infer a combined phylogenetic tree; and (ii) exploration of precomputed phylogenetic trees that integrate both functionally validated and uncharacterized entries across the database (Figs. 6A, 6B and S6A). To view a specific tree, users can search for a sample in the Search module, open its detailed entry page, and click the “Phylogenetic Tree” button. These two modes enable investigation of the evolutionary context of antibodies and the inference of potential functions through tree-based similarity. Tree files can be retrieved by antibody name, with visualizations available in PNG format. In addition, a corresponding annotation table summarizing mutation profiles and functional characteristics of related antibodies is provided, facilitating downstream analyses of antibody evolution under immune selection pressure. In the precomputed mode, we applied our phylogenetic pipeline to all MAAD entries with available nucleotide sequences (∼18,000 sequences), which were grouped into several hundred distinct clones. Within this large-scale clustering result, our pipeline successfully grouped the reported lineage members together, and the resulting tree topology closely matched that described in the original study (Zost et al. 2021) (Fig. S6B). This large-scale replication of a known lineage within thousands of entries demonstrates the accuracy and robustness of our sequence-based clustering pipeline. Furthermore, these phylogenetic trees provide insights into patterns of amino acid mutation and potential functional convergence within antibody families. For entries lacking direct functional evidence, phylogenetic proximity to well-characterized antibodies can offer indirect inferences regarding their target specificity and cross-reactivity. For example, within a clonally related group of SARS-CoV-2-targeting antibodies, ADI-75585 and ADI-75630 exhibited negligible neutralization activity against Omicron-BA.1, whereas others showed strong neutralization (Fig. S6B). These two antibodies were positioned at a greater phylogenetic distance from the neutralizing members, a pattern consistent with their observed functional profiles.

## Discussion

In this study, we developed MAAD, a multidimensional antiviral antibody database targeting pathogens from three major RNA viral families. The current version of MAAD includes 27,414 curated entries, each annotated with standardized metadata including amino acid and nucleotide sequences, V/J germline usage, CDRs, SHM, targeted antigens, functional annotations, and structural information (Table S1). In addition, MAAD incorporates clinically evaluated therapeutic antibodies, providing a unique benchmark for comparison and enabling translational insights derived from well-characterized and successful antiviral agents.

Compared with existing antibody databases, MAAD extends far beyond a data aggregation repository by providing unique analytical capabilities that enable multilayered exploration of antibody–antigen relationships. These capabilities are delivered through a suite of interactive analytical modules for in-depth analysis of antibody sequence characteristics, antigen–antibody complex interface features, and functional inference through tree-based similarity. The platform supports CDR annotation, V/J gene usage profiling, and both full-length and CDR-based similarity searches, complemented by visual tools such as germline dot plots and sequence logo diagrams. This integrative framework reveals both conserved and virus-specific patterns of germline usage, providing intuitive insights and a systematic reference for evaluating gene biases across antiviral responses. Collectively, these tools transform MAAD from a static repository into a dynamic, user-friendly platform for antibody repertoire profiling and the elucidation of sequence–function relationships.

Beyond sequence-level characterization, MAAD also integrates structural and virological dimensions by systematically mapping interface residues and annotating them with site-specific Shannon entropy and mutation frequency, thereby providing a more comprehensive resource with structural insights essential for understanding viral immune escape. The emergence of SARS-CoV-2 variants during the COVID-19 pandemic demonstrated the virus’s ability to evade vaccine or therapeutic antibodies, leading to breakthrough infections and reinfections (Carabelli et al., 2023; Zhang et al., 2024). For example, Regdanvimab exhibited neutralizing activity against multiple SARS-CoV-2 variants, including Gamma, Delta, Epsilon, and Kappa, but it has been demonstrated to have significant escape from Omicron variants (Planas et al., 2022). Structural analysis of the Regdanvimab-spike complex in MAAD highlights several key contact residues (K417N, E484A, Q493R, and Y505H) that display both high entropy and high mutation frequency (Fig. 4B). These residues have been proven to directly impact the antigen–antibody interface and correlate with the loss of neutralization potency (Cao et al., 2022). Similarly, in the case of RSV, clinical trials of the monoclonal antibody Suptavumab revealed complete loss of neutralization against circulating RSV-B strains due to two amino acid substitutions (L172Q and S173L) in the F protein (Simões et al., 2021). Moreover, recent studies have identified RSV-B variants carrying mutations at residues 206 and 209 in the F protein that confer reduced susceptibility to another monoclonal antibody, Nirsevimab, resulting in a 1.3- to 300-fold increase in IC_50_ values (Wilkins et al., 2023). These observations underscore the importance of elucidating molecular interactions between antibodies and viral antigens, which serve as the foundation for rational antibody therapeutic design and optimization. MAAD also distinguishes itself by integrating a unique module that supports phylogenetic clustering and tree construction for user-submitted sequences, as well as exploration of precomputed trees that integrate both functionally validated and uncharacterized antibodies. This module is particularly relevant in the context of NGS of antigen-responding B cell repertoires, which generates large-scale antibody sequences but often lacks direct experimental validation (Goldstein et al., 2019). Computational clustering and phylogenetic reconstruction therefore provide a valuable means to infer antibody specificity and functional potential from uncharacterized sequences. These trees, paired with mutational and binding annotations, support functional inference, enable detailed evolutionary analyses, and facilitate the identification of promising therapeutic leads through sequence-based clustering and functional annotation. Taken together, these modules distinguishing MAAD from existing antibody databases by transforming it from a static repository into an integrative analytical platform. As a result, MAAD delivers a comprehensive framework for understanding antigen recognition and providing actionable guidance for rational antibody design and optimization.

While MAAD primarily integrates qualitative functional annotations, rather than raw assay readouts, this design helps minimize the direct impact of experimental variability across studies. Functional annotations in the current version are classified as “binding” or “neutralizing” based on the results reported in the original studies. Nonetheless, we acknowledge that assay heterogeneity in experimental assays remains an inherent limitation. In future updates, MAAD will continue to expand to cover a broader spectrum of pathogens. It will incorporate more detailed experimental metadata, including quantitative parameters such as binding affinity (e.g., *K*_D_) and neutralizing potency (e.g., IC_50_), together with information on experimental methods and testing conditions. In addition, results from deep mutational scanning (DMS) (Fowler and Fields, 2014) will be integrated to provide comprehensive mutational landscapes that link sequence variation to changes in binding affinity and immune escape potential. Importantly, the standardized structure of MAAD entries makes it well-suited for AI-driven applications. Its curated integration of antibody sequence, structure, and function provides a robust foundation for machine learning. Features such as aligned full-length antibody sequences, variable regions, V/J gene assignments, and mapped structural binding residues serve as high-quality input data for deep learning models. These resources create a fertile training ground for machine learning models in tasks, such as paratope prediction, neutralization classification, and cross-reactivity forecasting, which are supported by experimentally validated data. Looking ahead, we plan to incorporate standardized NGS datasets from antigen-enriched B cell repertoires, particularly those with paired heavy–light chains and confirmed antigen specificity. Collectively, these enhancements will greatly extend the scope and the power of AI-driven discovery within MAAD, reinforcing its role as both a research resource and a platform for translational innovation. To support the continuous expansion of the system, it was developed using the Spring Boot framework with a MySQL database backend, following a standard three-tier architecture consisting of the presentation, service, and data access layers, which facilitates functional extension. The system achieved an average query response time below 0.5 s, demonstrating stable performance. The database schema is designed to allow flexible incorporation of new data types, such as the experimental metadata and DMS data mentioned above. Meanwhile, the system adopts a modular and layered design which supports distributed deployment, enabling horizontal scalability in future large-scale applications. To ensure long-term sustainability, MAAD is actively maintained by the core development team and scheduled for semiannual updates to incorporate newly published antibody sequences, structures, and functional annotations.

In summary, MAAD serves not only as a comprehensive antiviral antibody database, but also as a versatile and extensible platform for sequence–function–structure integration, thereby supporting rational therapeutic antibody design, and AI-assisted antibody discovery. Through the integration of large-scale curated data and versatile analytic platforms, MAAD provides a foundation for rational antibody design, therapeutic optimization, and AI-driven discovery, ultimately advancing preparedness against current and future viral threats.

## Supplementary Material

pwaf106_Supplementary_Data

## Data Availability

All the data in this research are available in MAAD (raabmd.org/raab/index).

## Code availability

The code used in this study is available from the corresponding author upon reasonable request.
