# Supplementary material for: MAAD: multidimensional antiviral antibody database
Source: Protein Cell. 2025 Dec 6;17(6):560–72. doi: 10.1093/procel/pwaf106 (PMC13225756; doi:10.1093/procel/pwaf106)
Supplement: pwaf106_Supplementary_Data [file pwaf106_supplementary_data.pdf]

Supplementary Materials for

MAAD: Multidimensional Antiviral Antibody Database

List of Supplementary Materials

Figure S1. Overview of entries in MAAD.

Figure S2. CDR3 length and SHM distributions of entries in MAAD.

Figure S3. Circos plots of germline gene pairings of antibodies.

Figure S4. Detailed page of MAAD showing.

Figure S5. Viral entropy and mutation analysis.

Figure S6. Workflow of sequence-based clustering and tree construction.

Table S1. Summary of fields in the MAAD database.

Figure S1. Overview of entries in MAAD.

(A) Donut plots showing the distribution of antibodies, nanobodies, and scFvs (left) and the developmental species origin of each entry in MAAD (right). (B) Polar bar plots summarizing the number of antibodies, nanobodies and scFvs annotated to bind to or neutralize each viral strain/subtype. *Coronaviridae*: SARS-CoV-2 (WT, Alpha, Beta, Gamma, Delta, Epsilon, Omicron), SARS-CoV-1 and MERS-CoV (right). *Pneumoviridae*: RSV-A, RSV-B, hMPV-A, and hMPV-B (middle). *Orthomyxoviridae*: Influenza A subtypes H1-H18 and influenza B (right). Bar lengths encode counts. Labels indicate the exact number of unique antibodies, nanobodies, and scFvs per category. Colors distinguish categories only.

Figure S2. CDR3 length and SHM distributions of entries in MAAD.

(A) and (B) are bar plots showing the distributions of heavy-chain (pink) and light-chain (blue) CDR3 lengths numbered by IMGT scheme and somatic hypermutation (SHM) counts of antibodies, nanobodies, and scFvs.

Figure S3. Circos plots of germline gene pairings of antibodies.

Circos plots showing the IGHV-IGHJ (heavy chain) and IGL/KV-IGL/KJ (light chain) gene pairings observed in antibodies targeting SARS-CoV-2, SARS-CoV-1, MERS-CoV, RSV, hMPV and influenza. Each arc represents a germline gene segment, and connecting ribbons indicate observed pairings between V and J genes. The width of the ribbons reflects the frequency of each pairing within the dataset. Only the top five IGHV and IGL/KV genes are shown.

Figure S4. Detailed page of MAAD showing.

(A) Search interface for querying entries and viewing detailed information, including binding/neutralization profiles, sequences, germline genes, CDRs and SHM. (B) Interactive interface of the CDR-based analysis module and the corresponding results, including selected CDR logo plots and SHM distribution plots.

Figure S5. Viral entropy and mutation analysis.

(A) Site-specific Shannon entropy calculated from the aligned HA sequences from H1N1, H3N2, H5N1, H7N9 and influenza B virus (Victoria and Yamagata lineages) to quantify sequence variability. (B) Bar plots display per-residue Shannon entropy (blue) and mutation frequency (orange) of RSV and hMPV G proteins, calculated based on aligned viral sequences relative to a reference strain. CT = Cytoplasmic tail; TM = Transmembrane; CCD = Central conserved domain. (C) Statistical comparison of Shannon entropy across different regions, including the RBD and remaining regions of the SARS-CoV-2 spike, the CCD and remaining regions of the RSV-A/B G protein, the mucin-like and remaining regions of the hMPV-A/B G protein and HA1 and HA2 of the influenza HA protein. Statistical significance was determined using Mann-Whitney tests (two groups) or Kruskal-Wallis tests with Bonferroni correction (multiple groups). (****$p < 0.0001$, *$p < 0.05$, ns: not significant)

Figure S6. Workflow of sequence-based clustering and tree construction.

(A) MAAD supports two modes of clustering analysis: (1) exploration of precomputed phylogenetic trees that incorporate both functionally validated and uncharacterized entries within the database and (2) the user-driven phylogenetic tree reconstruction, in which user-uploaded sequences are integrated with MAAD sequences to infer a combined phylogenetic tree. In user-driven modes, nucleotide sequences are first grouped by CDR3 length and pathogen source (optional). In both modes, clonal assignment is performed with Change-O, multiple sequence alignment is conducted using MAFFT, and phylogenetic trees are constructed with IQ-TREE and visualized using ETE3. (B) Examples of phylogenetic trees illustrating lineage clustering (left) and functional-phenotypic relationships (right).

Table S1. Summary of fields in the MAAD database	
Seq	Unique sequence identifier for Ab, Nb and scFv
Name	Published name of Ab, Nb and scFv
Nickname	Commonly used nickname of Ab, Nb and scFv
AborNb	Specifies whether the entry is an Ab, Nb or scFv
Virus	The virus from which B cells producing the Ab, Nb or scFv were derived
Bindsto	Antigens experimentally confirmed to bind to Ab, Nb and scFv
Doesnot Bindto	Antigens experimentally confirmed not to bind to Ab, Nb and scFv
NeutralisingVs	Antigens experimentally neutralized by Ab, Nb and scFv
Not NeutralisingVs	Antigens experimentally tested but not neutralized by Ab, Nb and scFv
Protein Epitope	Protein domain targeted by Ab, Nb and scFv
Origin	Developmental biological or synthetic origin of Ab, Nb and scFv (e.g. human, murine, engineered)
Species	Species of origin of Ab, Nb and scFv
VHorVHH	Heavy variable domain amino acid sequence
VH nuc	Heavy variable domain nucleotide sequence
VHGenbankID	Accession number of GenBank for the heavy variable domain nucleotide sequence
VL	Light variable domain amino acid sequence
VL nuc	Light variable domain nucleotide sequence
VLGenbankID	Accession number of GenBank for the light variable domain nucleotide sequence
Structures	Links to available antigen-Ab/Nb complex structures
PMID	PubMed identifier linking to the primary reference
Reference	References to the primary literature on Ab, Nb and scFv
Last updated	Timestamp indicating when the Ab, Nb and scFv was added
Pub date	Publication date of Ab, Nb and scFv
Heavy/Light identity species	Species of the most identical germline heavy or light chain
Heavy/Light V Gene	The identifier of the most sequence identical germline over the heavy or light chain v-region
Heavy/Light J Gene	The identifier of the most sequence identical germline over the heavy or light chain j-region
CDRH/L1, CDRH/L2, CDRH/L3 (imgt, kabat, chothia)	IMGT, kabat and chothia numbering for heavy and light chain CDRs
CDRH/L1_len,	CDR lengths of heavy and light chain (IMGT, kabat

CDRH/L2_len, CDRH/L3_len (imgt, kabat, chothia)	and chothia scheme)
Count_cdr1/2_r_heavy, Count_fwr1/2/3_r_light Count_cdr1/2_s_heavy, Count_fwr1/2/3_s_light	Mutation counts in CDR and FWR regions, binned as replacement (r) or silent (s)
Substitution_heavy, Substitution_light	Total number of replacement (r) and silent (s) mutations in CDR1/2 and FWR1/2/3

Figure S1

A

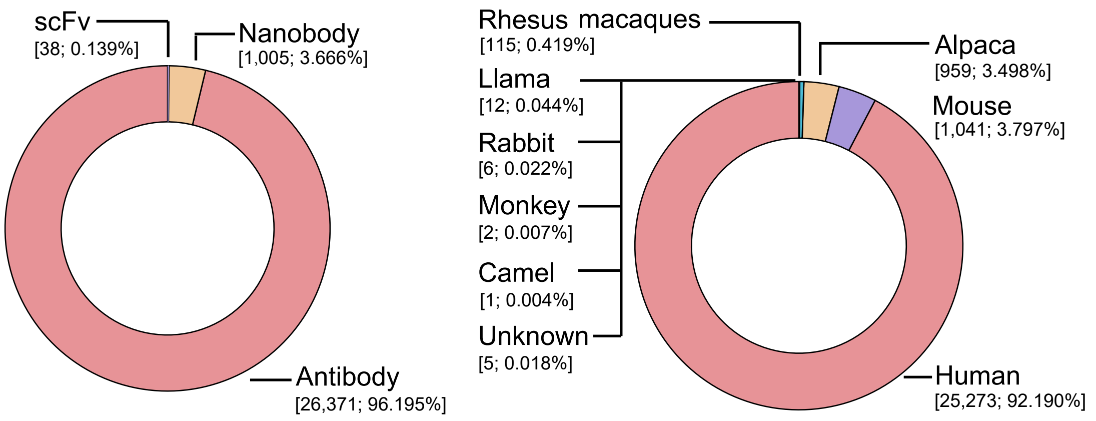

scFv
[38; 0.139%]
Nanobody
[1,005; 3.666%]
Antibody
[26,371; 96.195%]
Rhesus macaques
[115; 0.419%]
Llama
[12; 0.044%]
Rabbit
[6; 0.022%]
Monkey
[2; 0.007%]
Camel
[1; 0.004%]
Unknown
[5; 0.018%]
Alpaca
[959; 3.498%]
Mouse
[1,041; 3.797%]
Human
[25,273; 92.190%]

B

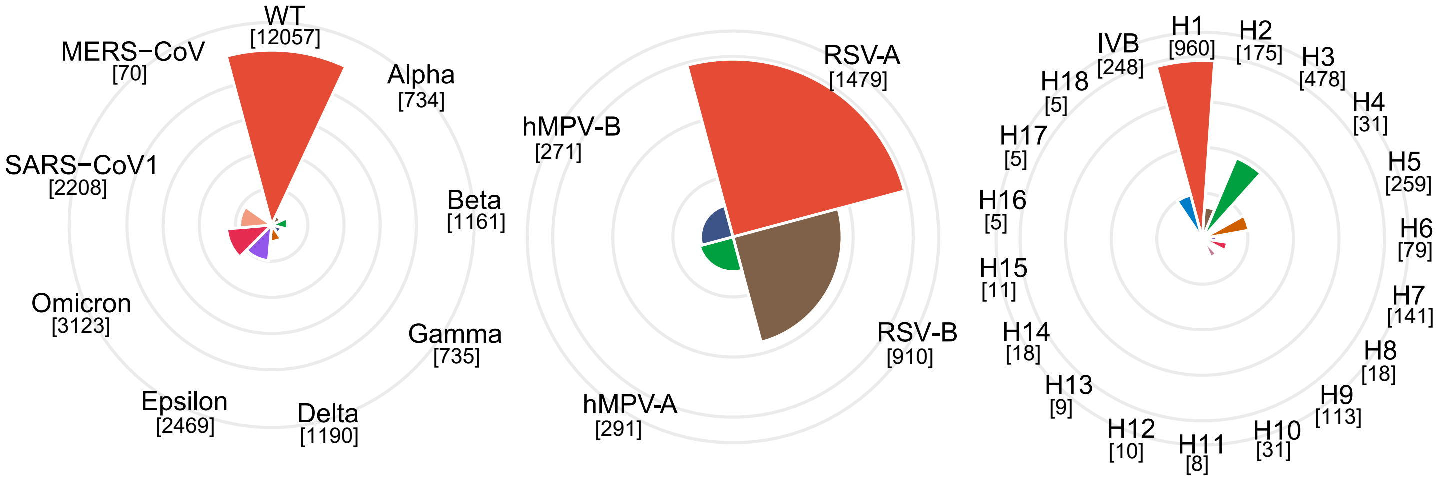

WT
[12057]
Alpha
[734]
Beta
[1161]
Gamma
[735]
Delta
[1190]
Epsilon
[2469]
Omicron
[3123]
SARS−CoV1
[2208]
MERS−CoV
[70]
RSV-A
[1479]
RSV-B
[910]
hMPV-A
[291]
hMPV-B
[271]
H1
[960]
H2
[175]
H3
[478]
H4
[31]
H5
[259]
H6
[79]
H7
[141]
H8
[18]
H9
[113]
H10
[31]
H11
[8]
H12
[10]
H13
[9]
H14
[18]
H15
[11]
H16
[5]
H17
[5]
H18
[5]
IVB
[248]

Figure S2

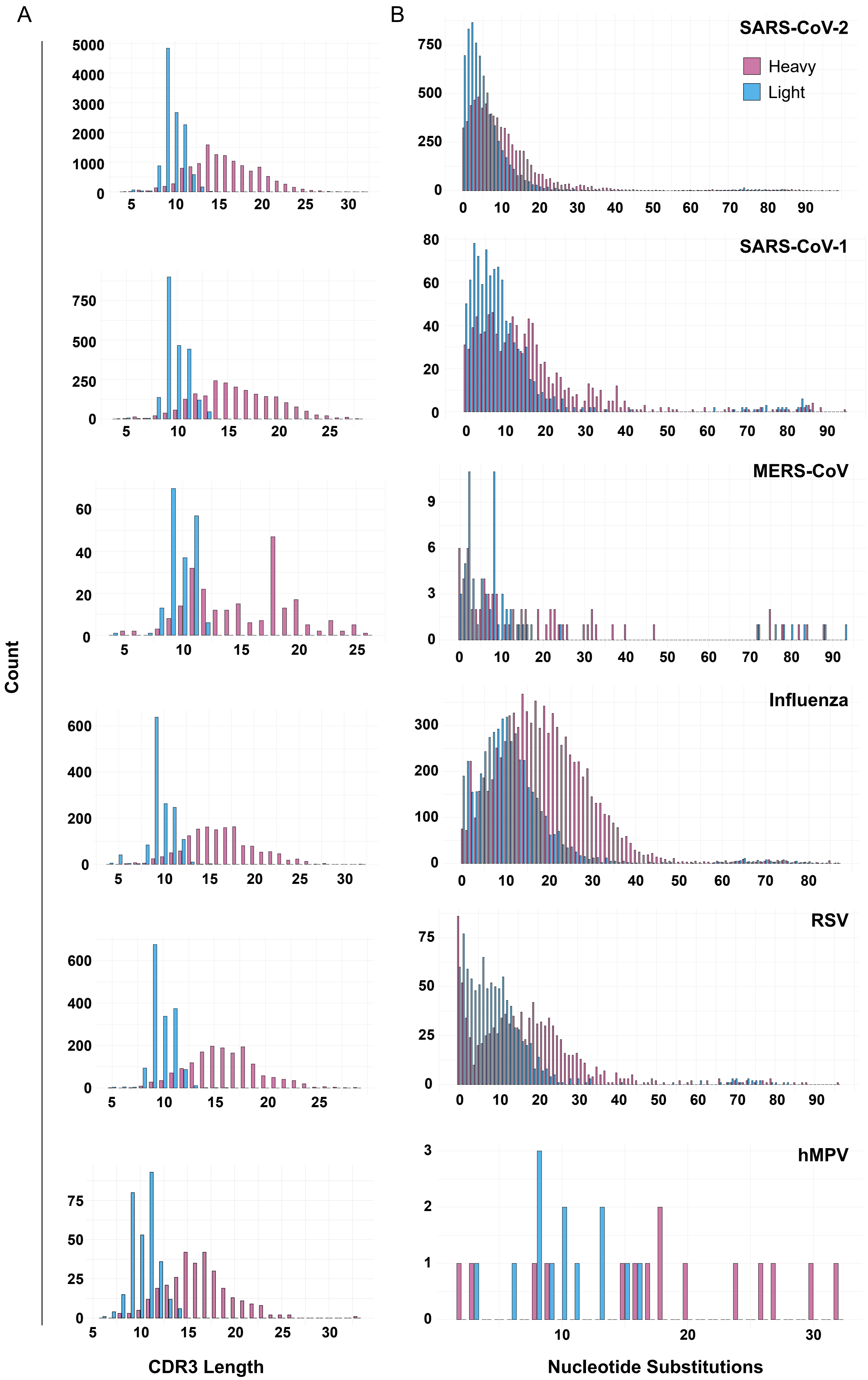

A
B
SARS-CoV-2
Heavy
Light
SARS-CoV-1
MERS-CoV
Influenza
RSV
hMPV
Count
CDR3 Length
Nucleotide Substitutions

Figure S3

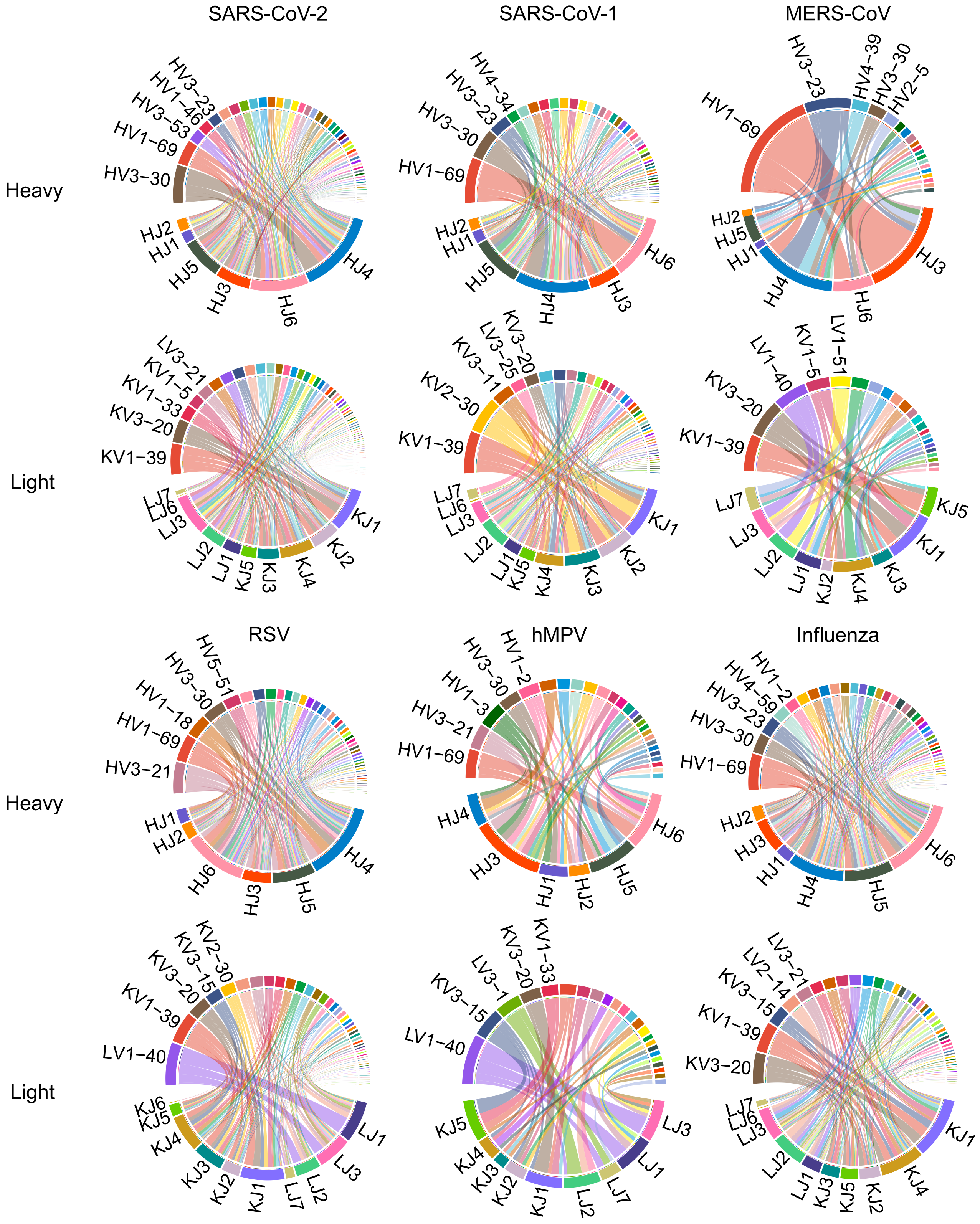
SARS-CoV-2
SARS-CoV-1
MERS-CoV
Heavy
Light
RSV
hMPV
Influenza
Heavy
Light

Figure S4

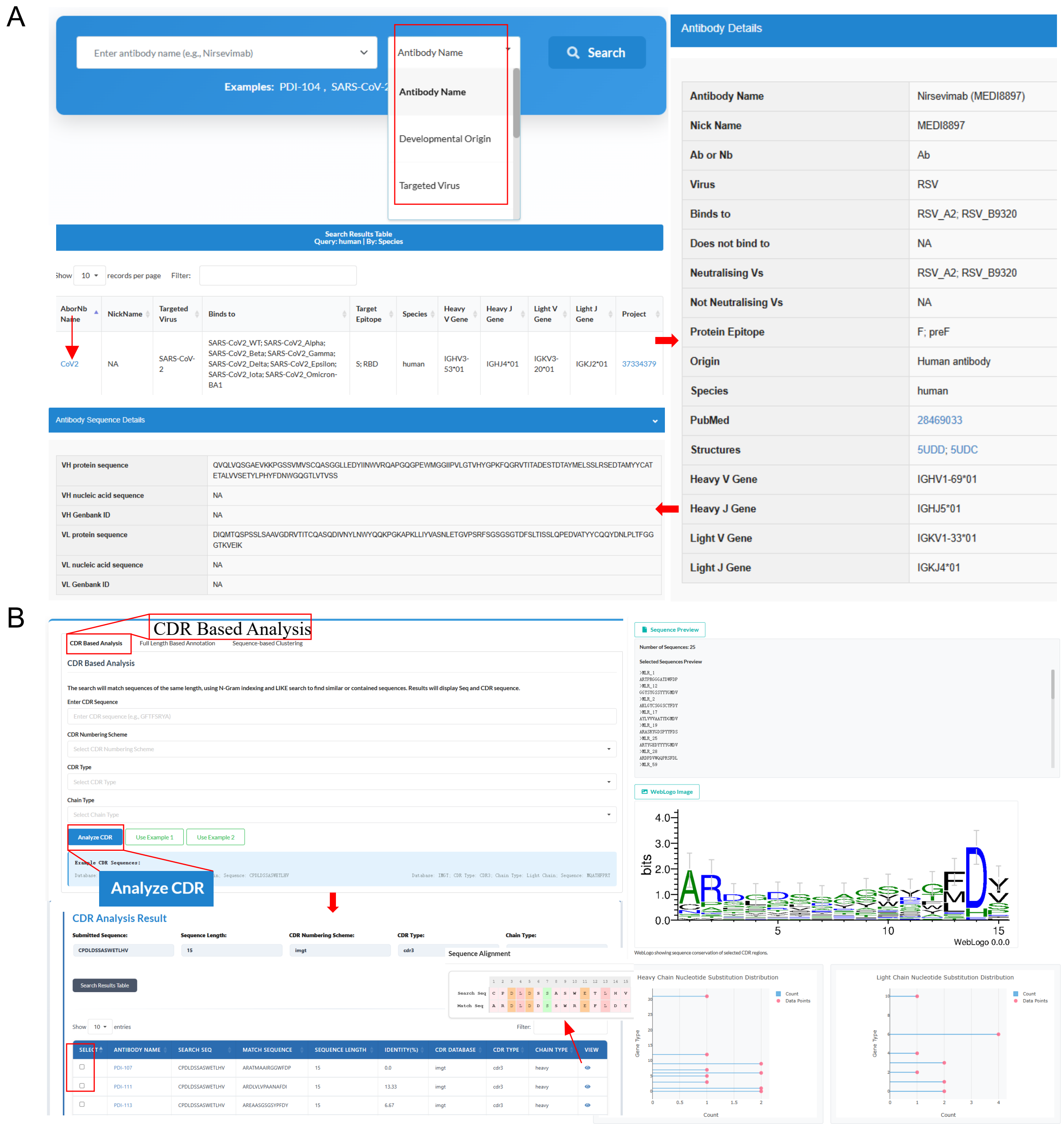
A
B
Antibody Details
Antibody Name
Nirsevimab (MEDI8897)
Nick Name
MEDI8897
Ab or Nb
Ab
Virus
RSV
Binds to
RSV_A2; RSV_B9320
Does not bind to
NA
Neutralising Vs
RSV_A2; RSV_B9320
Not Neutralising Vs
NA
Protein Epitope
F; preF
Origin
Human antibody
Species
human
PubMed
28469033
Structures
5UDD; 5UDC
Heavy V Gene
IGHV1-69*01
Heavy J Gene
IGHJ5*01
Light V Gene
IGKV1-33*01
Light J Gene
IGKJ4*01
Search
Antibody Name
Developmental Origin
Targeted Virus
CDR Based Analysis
Analyze CDR
CDR Analysis Result
WebLogo Image
Sequence Preview
Sequence Alignment
Heavy Chain Nucleotide Substitution Distribution
Light Chain Nucleotide Substitution Distribution

Figure S5

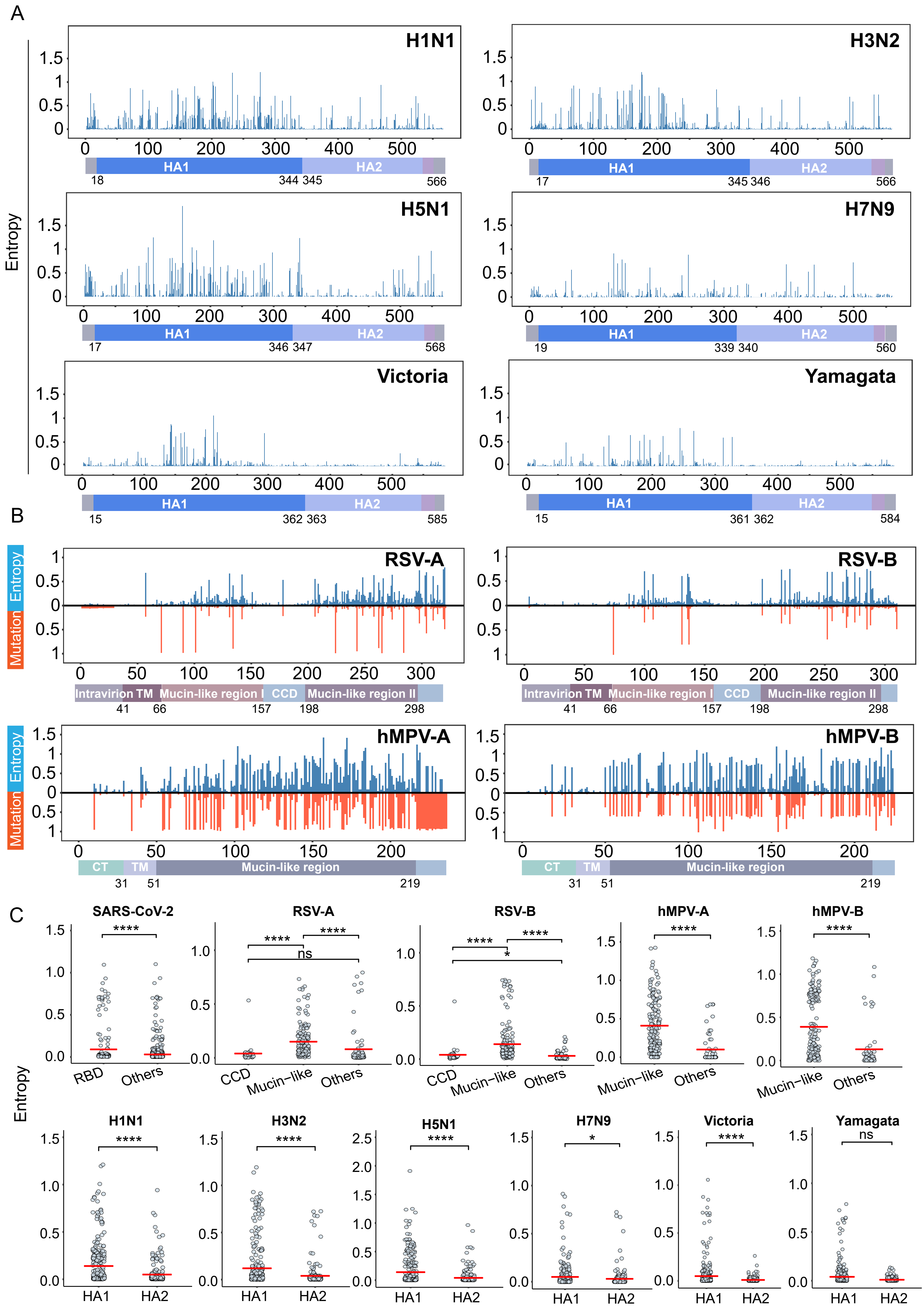
A
H1N1
H3N2
H5N1
H7N9
Victoria
Yamagata
Entropy
HA1
HA2
B
RSV-A
RSV-B
hMPV-A
hMPV-B
Entropy
Mutation
Intravirion
TM
Mucin-like region I
CCD
Mucin-like region II
CT
Mucin-like region
C
SARS-CoV-2
RBD
Others
Mucin-like
ns
****
*

Figure S6

A

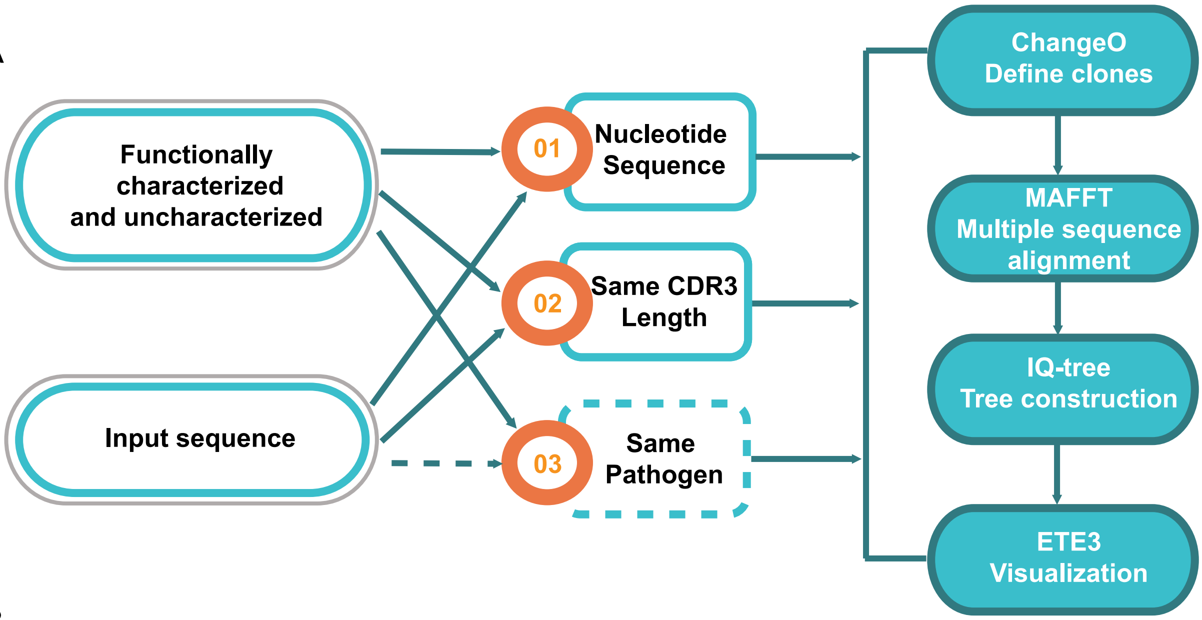

Functionally characterized and uncharacterized
Input sequence
01
Nucleotide Sequence
02
Same CDR3 Length
03
Same Pathogen
ChangeO Define clones
MAFFT Multiple sequence alignment
IQ-tree Tree construction
ETE3 Visualization

B

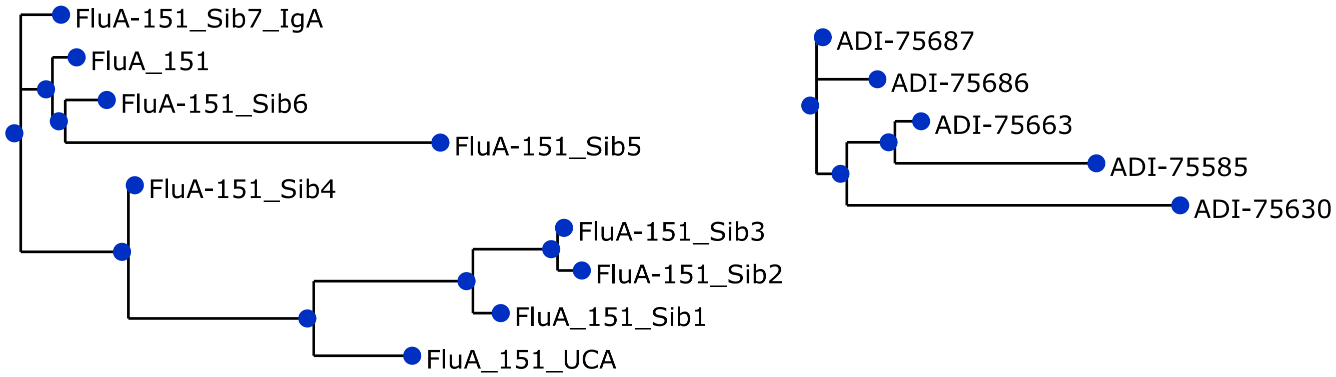

FluA-151_Sib7_IgA
FluA_151
FluA-151_Sib6
FluA-151_Sib5
FluA-151_Sib4
FluA-151_Sib3
FluA-151_Sib2
FluA_151_Sib1
FluA_151_UCA
ADI-75687
ADI-75686
ADI-75663
ADI-75585
ADI-75630

Methods

Data collection, processing, and curation

Antibody, nanobody, and scFv entries targeting coronaviruses (CoVs) were obtained from CoV-AbDab (Raybould et al. 2021). Entries targeting other pathogens were collected through keyword-based searches in PubMed, bioRxiv, GenBank (Benson et al. 2011), and Google Patents, using terms such as "RSV/hMPV/Influenza antibody", "RSV/hMPV/Influenza antibody profiling", and "RSV/hMPV/Influenza B cell response". A comprehensive set of references and patents was integrated into the database. For antibodies with resolved structures, we retrieved the structures from the Protein Data Bank (https://www.rcsb.org/) (Berman et al. 2000) by searching relevant keywords in the structure title. Therapeutic antibodies were collected from Thera-SAbDab (Dunbar et al. 2014) and PubMed. We additionally identified antibodies in clinical development by searching PubMed with the keyword "clinical trial" to capture candidates evaluated in clinical studies and to record their clinical stages.

All antibody amino acid and nucleotide sequences were obtained either directly from the original publications and patents or via GenBank using accession IDs cited in the source literature. For entries with available amino acid sequences, the heavy chain was required to be at least 100 amino acids in length and contain identifiable germline and CDR regions, while the light chain was required to be at least 90 amino acids with assignable germline and CDR annotations. In both chains, the number of missing residues could not exceed two. For entries lacking amino acid sequences, inclusion was limited to those with at least one of the following: a functional annotation (binding or neutralization), reported CDR sequences, or germline gene assignments. Full-length variable region sequences were processed using ANARCI (Dunbar and Deane 2016) to annotate CDRs and assign corresponding V/J germline genes, which employed Hidden Markov Models to align input sequences to pre-numbered germline references. CDRs were annotated based on three standardized numbering schemes: the international IMGT (Lefranc et al. 2003), Kabat (Kabat and Wu 1971), and Chothia (Chothia and Lesk 1987). To assess SHM, germline-aligned sequences were further processed using the Change-O toolkit (Gupta et al. 2015) to generate detailed germline alignment data. SHM frequencies were quantified with the *observedMutations* function implemented in the R package shazam (Gupta et al. 2015), which calculates the number of nucleotide differences between each antibody sequence and its inferred germline reference across the framework 1/2/3 and CDR 1/2 regions.

Viral genome sequence collection

Viral genome sequence data were collected from Nextstrain (https://nextstrain.org/) (Hadfield et al. 2018) and the NCBI (NCBI Resource Coordinators 2017). Preliminary sample metadata were first downloaded from Nextstrain, and the strain names were extracted to facilitate sequence retrieval from NCBI. To minimize potential data loss, the NCBI Datasets command-line interface (CLI) was additionally used for direct sequence acquisition. Genome sequences were retrieved using taxon-specific keyword queries. For example, MERS-CoV genomes were obtained with the

带格式的: 左

command: *datasets download virus genome taxon* "*Middle East respiratory syndrome coronavirus*". Subsequently, the associated metadata were extracted using the command: *dataformat tsv virus-genome*. These procedures were applied for each target virus to generate a standardized dataset of genome sequences and corresponding metadata for downstream analyses.

Antigen-antibody complexes interface residue analysis

For each antigen-antibody complex, the antigen chains and the corresponding antibody heavy and light chains were retrieved from SAbDab (Dunbar et al. 2014). To characterize antigen-antibody interfaces, an in-house Python script was used to identify interface residues. Interface residues were defined as residues on the antigen and antibody partner chains with any interatomic distance of less than 4.5 Å. Each interface residue was annotated at the site level with entropy and recorded amino acid substitutions relative to a reference sequence.

Normalized Shannon entropy analysis

After data collection, BLAST (v2.16.0) (Altschul et al. 1990) was used to compare each viral sequence against its corresponding reference genome in order to remove low-quality sequences. Sequences were filtered using two criteria: (i) only alignments covering ≥ 80% of the reference genome were retained, and (ii) sequences with BLAST identity ≥ 80% were kept. The reference genomes used for each virus were: MERS-CoV (MF598664), SARS-CoV (NC_004718), SARS-CoV-2 (MN908947), RSV type A (PP109421), RSV type B (OP975389), hMPV type A (NC_039199) and hMPV type B (AY525843). The reference HA genes used for influenza virus were: H1N1 (AFM72832), H3N2 (AHG96407), H5N1 (AAD51927), H7N9 (AHK10800), Victoria strain (ANC28539) and Yamagata strain (AET22022). Sequences passing these filters were subjected to multiple sequence alignment using MAFFT (v7.525) (Katoh et al. 2002). To quantify sequence variability at each position, we calculated the normalized Shannon entropy: $H_i = -\sum_{a \in A} P_i(a)\, ln\, P_i(a)$ where H_i is the entropy at alignment position *i*, *A* denotes the set of amino acids observed at that position, and $P_i(a)$ is the relative frequency of amino acid *a,* defined as $P_i(a)$ = (*number of sequences containing residue a at position i*) / *(total number of sequences at position i*). This formulation accounts for all possible residues observed at a given site.

Mutation per-site analysis

Mutation frequencies were calculated based on multiple sequence alignments. For each alignment position *i*, the reference residue was recorded, and all residue occurrences (including the reference) at that position were counted across all sequences. Gaps were excluded from the counts. For each observed residue *a*, the mutation frequency was defined as $f_i(a) = \frac{n_i(a)}{N}$, where $n_i(a)$ is the number of sequences containing residue *a* at position *i*, and *N* is the total number of sequences in the alignment.

Phylogenetic clustering of antibody sequences

For a given set of antibody heavy chain nucleotide sequences, we first applied ANARCI (IMGT scheme) (Dunbar and Deane 2016) to extract the CDR3 region. To enable phylogenetic clustering, the antibody database was filtered to retain only

entries containing nucleotide information and having the same CDR3 length as the input sequence. Sequences passing these filters were processed with Change-O (v1.3.3) (Gupta et al. 2015) and used Hamming distance to define clonal groups. For each clone, all assigned sequences were aligned using MAFFT (v7.525) to generate multiple sequence alignments. These alignments were subsequently used as input to IQ-TREE (v2.2.2.6) (Nguyen et al. 2015). IQ-TREE was instructed to perform an extended model selection using ModelFinder Plus (MFP), invoked with the option -m MFP, which automatically identifies the most appropriate substitution model for the data and constructs the tree under that model. The resulting phylogenetic trees were further processed and visualized using ETE3 (Huerta-Cepas et al. 2016), a Python package for tree manipulation and visualization. In addition to tree construction, mutations were extracted from each internal node to characterize evolutionary changes along the branches. Furthermore, functionally characterized and uncharacterized sequences in the database were combined and pre-clustered using the same phylogenetic clustering strategy described above.

Database implementation

The database was constructed using a modular, three-tier web architecture to enable efficient curation, querying, and visualization of antibody sequence variants and their functional implications. The backend is implemented with Spring Boot 3.4.4, providing a robust foundation for server-side logic, RESTful API design, and request handling through Java Servlets. Dynamic content rendering is achieved using the Thymeleaf template engine, which integrates seamlessly with HTML-based views to deliver data-driven pages. All curated antibody data, including variable domain sequences (VH and VL), germline gene assignments, CDRs, somatic hypermutations, and experimentally validated functional variants are stored in a structured MySQL relational database. The database schema is normalized to minimize redundancy and ensure data consistency, with indexed fields on key identifiers such as antibody names, antigen targets, and mutation positions to optimize search performance. MyBatis is employed as the persistence layer to map Java objects to database records, supporting flexible and high-performance querying for complex use cases such as CDR-based filtering, mutation co-occurrence analysis, and lineage tracking. The frontend interface is built with HTML5, CSS3, and JavaScript, and enhanced with jQuery 3.6.0 for DOM manipulation, Semantic UI 2.5.0 for responsive and accessible user interface components, and DataTables 1.11.5 for interactive tabular display with sorting, pagination, and keyword search. To support structural interpretation of antibody-antigen interactions, the database integrates PDBe Molstar 3.3.0, a WebGL-powered molecular viewer, enabling users to visualize 3D structure of Fab or Fv fragments and explore the spatial context of mutations within antigen-binding sites directly within the browser.
